# Hospitals and Medical Education

**Published:** 1908-07-25

**Authors:** 


### The Hospital

A Journal of

The Medical Sciences and Hospital Administration.

New Series. No. 72, Vol. III. [No. 1144, Vol. XLIV.] SATURDAY, JULY 25, 1908.

### Hospitals and Medical Education.

It has for a very long time been evident that the changes brought about in the methods of teaching and training medical students by the development of scientific methods during the past half century have rendered obsolete and impossible the continuation of the London medical schools, which alone are as yet unendowed, on their traditional lines. The signs of the times, whose tendency has been observed for some years now, have at last become so obvious that they can no longer be neglected, and it is as clear as daylight that some reconstruction of the edifice of medical education in London is essential. As to the exact details of how this task is to be attempted, opinions, even among the elect, unfortunately differ very markedly, and these differences have within the last few days come to a head in a discussion in the columns of the *Times*.

Let us consider briefly what is the malady for which different remedies are suggested by the faculty. The main cause which has modified the working of the various schools is the enormously increased cost of teaching, owing to the development of laboratory methods of investigation and instruction. Even to teach a class clinical medicine in a ward involves the constant use of bacteriology, blood examination, electrical appliances, and what not; while these subjects and many others equally costly have taken their place among the necessities of the preliminary instruction. In physiology, as well as in pathology, the same process is going on, and it has come to this, that medical schools cannot maintain the standard of twentieth century efficiency as such, except by raising their fees to prohibitive heights or by seeking financial endowment from some other source. The problem is further complicated by the prohibition which is levelled at all the metropolitan hospitals by the King's and the Hospital Sunday Funds, forbidding them to contribute from their revenue to an attached medical school unless their income contains items in equal amount earmarked by the donors for that purpose.

One way of dealing with a concern which is approaching bankruptcy is to reduce its expenses; but the difficulty in the London schools is to do anything of the sort, for the salaries—when they exist, which is not always—of the teachers are already miserably inadequate, and laboratory expenses with each new discovery increase instead of diminishing. Another suggestion having the same end in view was that which came to grief last year, the proposal to found an Institute of Medical Sciences in connection with London University, at which all the preliminary studies should be carried out. The enormous saving and the increased tutorial efficiency possible by a well-arranged scheme on these lines, compared with the present wasteful system is self-evident; but for various reasons, which we considered in these columns on July 13 last year, the particular scheme fell through and was abandoned. It was the more regrettable that something useful and permanent did not come of the attempt, because very considerable sums of money—£100,000 in all—were subscribed or promised to endow the Institute, and within the past week the executors of Mr. Beit have had to ask a Court of Law to decide on the correct and legal distribution of £25,000 which he bequeathed for the same purpose.

The only other way of utilising as they should be used the unrivalled clinical materials which London provides, is to raise separate endowment funds for individual schools, or a central fund which shall distribute to the latter; and this solution of the problem is powerfully advocated by the President of the Royal College of Surgeons in a letter published in the *Times* on July 12. In this letter Mr. Morris permitted himself to allude in disparaging terms to the rejected scheme of last year, which was the more inadvisable because the reasons for its non-acceptance were accidental rather than unavoidable, extraneous rather than inherent. The result of those allusions was, not unnaturally, to provoke into recrimination advocates of the institute scheme, men, be it noted, of as great eminence and ability as Mr. Morris himself. Into these differences of opinion there is no need for us to intrude; for our part we welcome any plan which seems to have a chance of saving the London schools from their embarrassments, and we are willing to applaud any whole-hearted endeavour to put them on the footing they occupied say twenty years ago. At the same time it must not be forgotten that a similar appeal was issued not long ago by the St. George's Hospital School, to which the response was distinctly disappointing; it remains to be seen whether the Middlesex School will have better luck. There is, it must be remembered, another aspect of the question, that of the State, which cannot be indifferent to any condition of affairs that shall block the avenues of approach to the profession whose future efficiency concerns it more vitally than any other. If medical education has become so expensive that only the wealthy can afford it, and if in those circumstances adequate assistance and endowment be not forthcoming, the result must be a shortage of the supply of doctors in the next generation, and this is a prospect to which no statesman who values his reputation can afford to shut his eyes.

